# Purified human anti-Tn and anti-T antibodies specifically recognize carcinoma tissues

**DOI:** 10.1038/s41598-019-44601-9

**Published:** 2019-05-30

**Authors:** Natacha Zlocowski, Veronica Grupe, Yohana C. Garay, Gustavo A. Nores, Ricardo D. Lardone, Fernando J. Irazoqui

**Affiliations:** 1 Centro de Investigaciones en Química Biológica de Córdoba, CIQUIBIC, CONICET and Departamento de Química Biológica Ranwel Caputto, Facultad de Ciencias Químicas, Universidad Nacional de Córdoba, Ciudad Universitaria, X5000HUA Córdoba, Argentina; 2 Fundacion para el Progreso de la Medicina, Laboratorio de Alta Complejidad, 9 de Julio 941, 5000 Córdoba, Argentina; 3 Present Address: Universidad Nacional de Córdoba, Facultad de Ciencias Médicas, Centro de Microscopía Electrónica, Bv. De la Reforma y Enfermera Gordillo, Ciudad Universitaria, 5016 Córdoba, Argentina

**Keywords:** Protein purification, Glycobiology, Tumour biomarkers

## Abstract

Described in several epithelial cancer cells, Tn- (GalNAcα1-O-Ser/Thr) and T- (Galβ3GalNAcα1-O-Ser/Thr) antigens are examples of tumor-associated antigens. Increased expression of Tn- and T-antigens is associated with tumor invasion and metastasis, and patients with high concentration of anti-Tn and anti-T antibodies have a more benign evolution of pathology. Asialofetuin (ASF) and ovine submaxillary mucin (OSM) are two glycoproteins that expose T- and Tn-antigen, respectively. In this work, using ASF or OSM we affinity-purified anti-T and anti-Tn antibodies from normal human plasma and tested their ability to specifically recognize tumor human tissues. Whereas purified anti-T antibodies (purity degree increase of 127-fold, and 22% recovery) were mainly IgG, for purified anti-Tn antibodies (purity degree enhancement of 125-fold, and 26% yield) the IgM fraction was predominant over the IgG one. IgG2 subclass was significantly enriched in both purified antibody samples. Purified antibodies did not bind normal human tissue (0/42), although recognized malignant tissues from different origin such as colon carcinoma (11/77 by anti-Tn; 7/79 by anti-T), breast carcinoma (10/23 by anti-Tn; 7/23 by anti-T), and kidney carcinoma (45/51 by anti-Tn; 42/51 by anti-T). Our results suggest that purified human anti-Tn and anti-T antibodies have a potential as anti-tumor therapeutic agents; restoring their levels in human sera could positively affect the evolution of patients with epithelial tumor pathologies.

## Introduction

The phenotype of epithelial cancer cell is greatly conditioned by glycoconjugates from glycoproteins, glycolipids and glycosaminoglycans. These terminal glycans are relevant in the cell-cell and cell-extracellular matrix communication, and critical points in the cancer cell invasion, proliferation and dissemination processes^1^. O-GalNAc glycans are a type of protein post-translational modification significantly affected in epithelial cancer cells^2^. In polymeric biosynthesis of O-GalNAc glycans, the first step occurring is the covalent linkage of N-acetylgalactosamine (GalNAc) to selected Ser/Thr residues of the acceptor protein to yield GalNAcα1-O-Ser/Thr (Tn-antigen), a reaction catalyzed by polypeptide-N-acetylgalactosaminyltransferases (ppGalNAc-Ts)^3^. The second monosaccharide linked to GalNAcα1-O-Ser/Thr may be galactose (Gal) or N-acetylglucosamine (GlcNAc), to generate core 1 glycan (Galβ3GalNAcα1-O-Ser/Thr, also called T-antigen), or core 3 glycan (GlcNAcβ3GalNAcα1-O-Ser/Thr), respectively. T-antigen biosynthesis involves Core 1 β3Gal-T (C1GalT), an ubiquitous enzyme found in most mammalian cells. Core 3 glycans are predominant in colonic and salivary mucins, where Core 3 β3GlcNAc-T catalyzes their biosynthesis. The β6-GlcNAc-T action on T-antigen and core 3 glycans yield core 2 and core 4 glycans, respectively. Galβ3/4GlcNAc units give rise to the backbone region of O-GalNAc glycans. Fucose and N-acetylneuraminic acid are frequent capping residues in these regions^4^.

O-GalNAc glycans present on carcinoma cells are commonly truncated structures exposing cryptic regions that are normally hidden. Tumor associated-antigens (TAAs) are terminal residues chemically well know with more often in cancer cells than normal cells. Tn- and T-antigens are examples of TAAs described in several epithelial cancer cells^5^. The increased expression of T- and Tn-antigens is associated with tumor invasion and metastases^6^.

Normal human sera contain multiple antibodies recognizing specific glycan residues^7^, and different hypothesis attempt to explain the origin of natural anti-glycan antibodies^8^. Natural anti-Tn and anti-T antibodies are present in normal human sera^9^, and studies of anti-Tn and anti-T antibodies in patients with epithelial carcinomas showed reduced levels of these anti-glycan antibodies^10^. In addition, pathology evolution of patients with high concentration of anti-Tn and anti-T antibodies is more benign^11^. These results suggest that restitution of human anti-Tn and anti-T antibodies should positively affect the evolution of patients with epithelial tumor pathologies.

Immunotherapy modulates the host’s immune response to TAAs, eradicates cancer cells by reducing host tolerance to TAAs and provides protection against the disease^12–14^. Passive immunotherapies, like monoclonal antibodies or engineered T-cell based therapies, are targeted to tumor cells by recognizing TAAs. Several immunotherapy strategies have been tested for anti-tumor responses using monoclonal antibodies against receptor tyrosine kinases like members of the EGFR family (cetuximab, pertuzumab, and trastuzumab)^15,16^ or against their ligands like VEGF (bevacizumab)^17^, involved in tumor cell proliferation or angiogenesis, respectively.

In the present study we purified two populations of antibodies (anti-Tn and anti-T) from pooled human plasma and evaluated their ability to recognize human carcinoma tissue, aiming to uncover potential applications in antineoplastic therapy.

## Results

### Purification of anti-glycan antibodies

**Figure 1:**
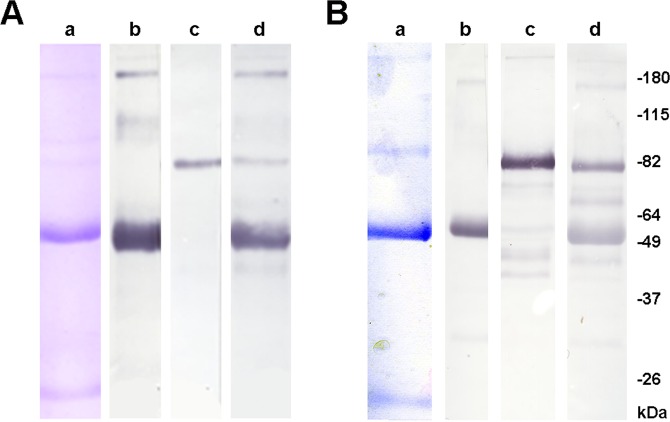
Analysis of purified proteins obtained by affinity chromatography using immobilized ASF (**A**) or OSM (**B**) antigen. Quadruplicates for each eluted sample were seeded in polyacrylamide gels to analyze degree of protein purity by staining with Coomassie Brilliant Blue (a), or by western blot detecting heavy chain presence of human IgG (b), human IgM (c), or human total immunoglobulins (d) by using HRP-labeled IgG anti-human IgG, or IgM or total immunoglobulins antibodies, respectively. Color reaction was developed as described in Methods.

Asialofetuin (ASF) and ovine submaxillary mucin (OSM) are two highly glycosylated antigens. ASF mainly exposes terminal T-antigen glycans, whereas OSM shows multiple terminal Tn- and sialyl Tn-antigens^18,19^. By immobilizing ASF and OSM in Sepharose, these terminal glycans were used as ligands for affinity chromatography purification of human antibodies. Gammaglobulin fraction from pooled human plasma was offered to immobilized antigens. After washing the columns, retained proteins were eluted and analyzed. The purity of eluted proteins was evaluated by SDS-PAGE stained with Coomassie Brilliant Blue (CBB), and immunoglobulin isotypes were identified by western blot (WB) using anti-human IgG, IgM and total immunoglobulin antibodies (Fig. 1).

**Figure 2:**
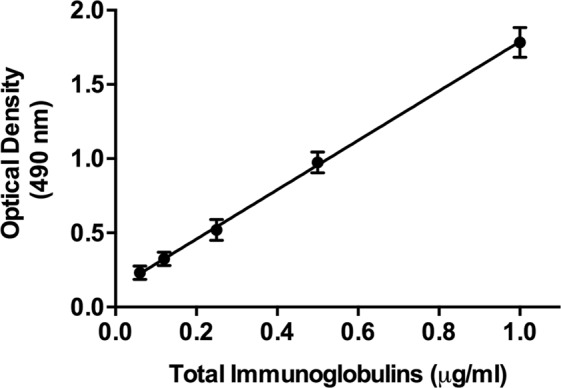
Measurement of total human immunoglobulin by ELISA. Standard curve was developed using different concentrations of purified human total immunoglobulins (Gammaglobulina-T, Laboratorio de Hemoderivados) adsorbed to wells of microtiter plates and detected with HRP-labeled IgG anti-total human immunoglobulin antibodies. Color reaction was developed as described in Methods. Concentration of total human immunoglobulin from different samples was measured extrapolating each optical density to concentration on the standard curve.

SDS-PAGE stained with CBB showed highly purified proteins, with molecular weight corresponding to human immunoglobulins. WB showed that purified anti-ASF antibodies are mainly IgG with reduced proportion of IgM, whereas in the purified human anti-OSM antibodies the IgM fraction is important with respect to IgG. To know the purification degree and yield of each purified antibody, proteins were measured by bicinchoninic acid assay, and human immunoglobulins were measured by ELISA using HRP-labeled IgG anti-human total immunoglobulin antibodies (Fig. 2). Affinity chromatography for human antibodies recognizing ASF increased the purity degree 127-fold with 22% recovery (Table 1), whereas the purification of anti-OSM antibodies enhanced 125-fold the specific activity with 26% yield (Table 2). Further characterization of both purified antibodies showed a very important enrichment of IgG2 subclass (Tables 1 and 2).

**Table 1:** Purification of anti-ASF antibodies containing anti-T antibodies from normal human plasma.

	Total proteins (µg)	Anti-ASF Abs (µg)	Specific Activity (anti-ASF Abs/proteins)	Purification (-fold)	Yield (%)	IgG/IgM ratio	IgG subclass (%)			
							1	2	3	4
Initial Igs	500,000	271	5.4 × 10^−4^	1	100	10	65	25	7	3
Eluted Igs	871	60	0.069	127	22	14	nd	100	nd	nd

nd: not detected.

**Table 2:** Purification of anti-OSM antibodies containing anti-Tn antibodies from normal human plasma.

	Total proteins (µg)	Anti-OSM Abs (µg)	Specific Activity (anti-OSM Abs/proteins)	Purification (-fold)	Yield (%)	IgG/IgM ratio	IgG subclass (%)			
							1	2	3	4
Initial Igs	500,000	425	8.5 × 10^−4^	1	100	10	65	25	7	3
Eluted Igs	1,025	109	0.106	125	26	0.8	nd	83	17	nd

nd: not detected.

### Carbohydrate specificity of purified anti-glycan antibodies

**Figure 3:**
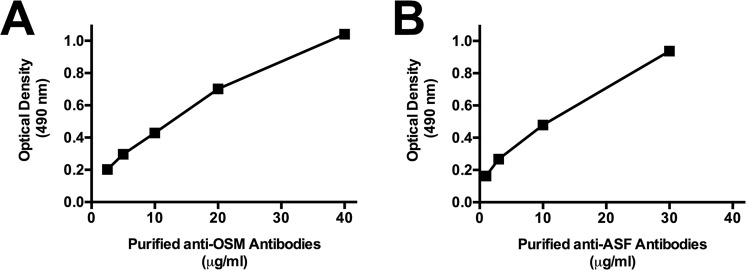
Recognition of purified antibodies to antigens by ELISA. Several concentrations of antibodies purified by affinity chromatography were faced to the corresponding ASF (**A**) or OSM (**B**) antigen adsorbed to wells of microtiter plates. After incubated for 2 h at RT, the interacting antibodies were detected with HRP-labeled IgG anti-total human immunoglobulin antibodies. Color reaction was developed as described in Methods.

The recognition of purified anti-ASF and anti-OSM antibodies to the corresponding antigen used during the affinity chromatography purification are shown in Fig. 3. Carbohydrate specificity of purified antibodies was evaluated by competitive ELISA using sugars related to Tn- and T-antigen. Interaction of anti-ASF antibodies was significantly inhibited with Galβ3GalNAcαBzl, whereas glucose did not induce inhibition (Table 3). Binding inhibition with Galβ3GalNAcαBzl revealed the presence of anti-T antibodies in this purified antibody sample. OSM-affinity chromatography yielded anti-Tn antibodies, as showed by the important inhibition with GalNAcαBzl whereas control (glucose) did not show inhibition (Table 4).

**Table 3:** Carbohydrate recognition of anti-T antibodies by competitive assay^1^.

Carbohydrate Inhibitor (%)	6 (mM)	3 (mM)	1,5 (mM)	0,75 (mM)
Galβ3GalNAcαBzl	39	24	15	5
Glc	6	5	3	4

^1^Competitive assay using carbohydrates as inhibitors of anti-ASF antibody recognition. Different concentration of carbohydrates preincubated with anti-ASF antibodies showed the significance of sugar residue in the antigen recognition. Galβ3GalNAcαBzl, structure related to T-antigen, is important inhibitor of antibody interaction whereas glucose (Glc) has not effect (control).

**Table 4:** Carbohydrate recognition of anti-Tn antibodies by competitive assay^1^.

Carbohydrate Inhibitor (%)	6 (mM)	3 (mM)	1,5 (mM)	0,75 (mM)
GalNAcαBzl	32	33	31	5
Glc	4	2	1	2

^1^Competitive assay using carbohydrates as inhibitors of anti-OSM antibody recognition. Different concentrations of carbohydrates preincubated with anti-OSM antibodies showed the significance of sugar residue in the antigen recognition. GalNAcαBzl, structure related to Tn-antigen, is important inhibitor of antibody interaction whereas glucose (Glc) has not effect (control).

### Anti-Tn and anti-T antibodies specifically recognize human carcinoma tissues

**Figure 4:**
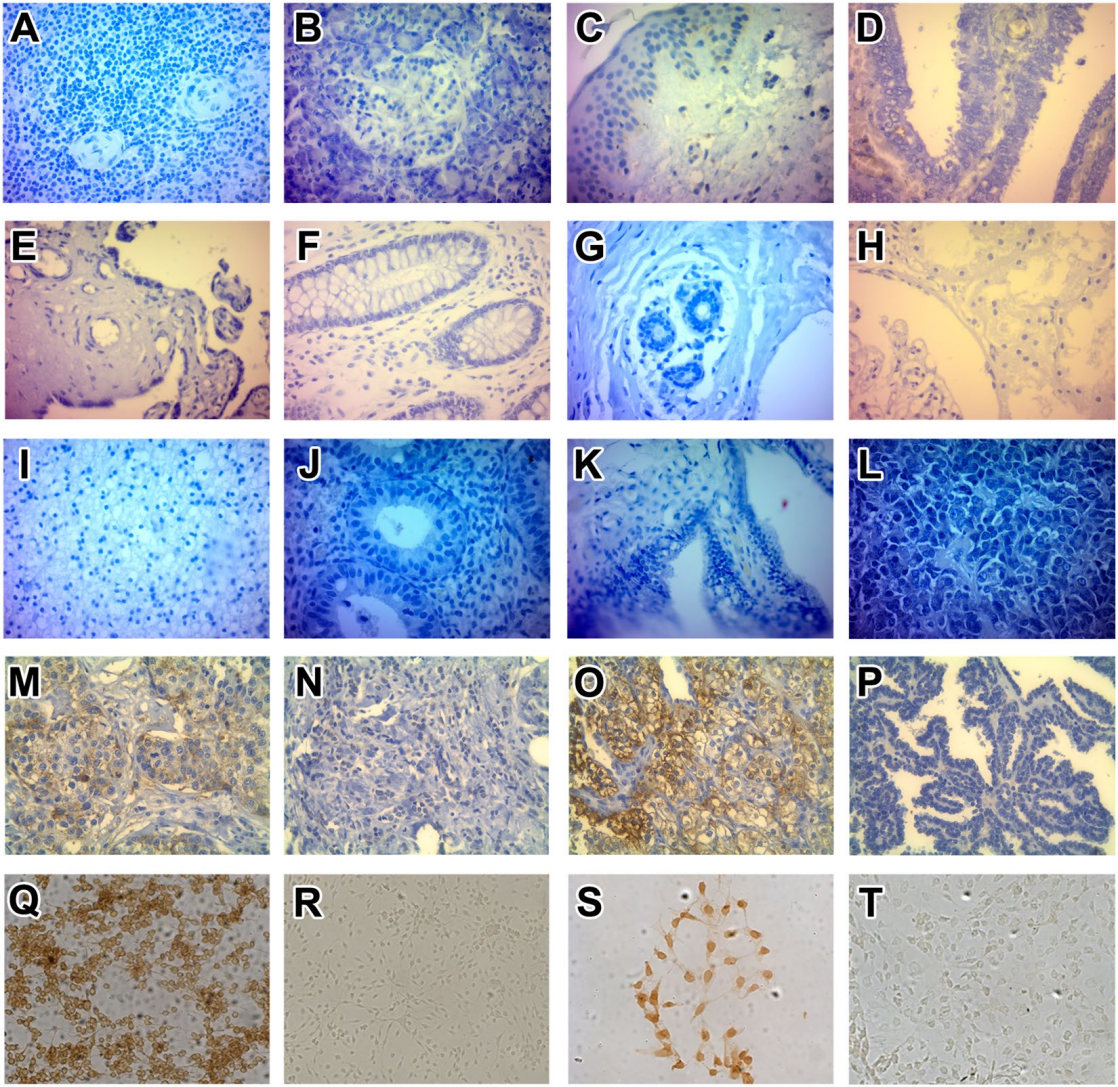
Representative examples of human normal and carcinoma tissues, and human cell lines in the analysis of anti-Tn and -T antibody recognition. No recognition (−) and positive recognition (+, brown color) of different human tissues/cells was observed by using human purified anti-Tn and -T antibodies. After incubated the TMAs/cells with primary anti-Tn or -T antibodies for 2 h at RT, interacting antibodies were detected with HRP-labeled IgG anti-total human immunoglobulin antibodies. Color reaction was developed with H_2_O_2_ and diaminobenzidine (brown color), using hematoxylin for contrast (blue color) in TMAs, as described in Methods. Human normal spleen (**A**), pancreas (**B**), skin (**C**), small intestine (**D**), placenta (**E**), colon (**F**), breast (**G**), kidney (**H**), brain (**I**), cervix (**J**), fallopian (**K**) and hypophysis (**L**) showed negative recognition by anti-Tn antibodies. Representative examples of human breast carcinoma tissues with positive recognition (**M**) and no recognition (**N**) by anti-T antibodies as well as positive recognition (**O**) and no recognition (**P**) to human kidney carcinoma tissues. Human breast cancer T47D cells show positive staining with anti-Tn antibodies (**Q**) and negative staining without the primary anti-Tn antibodies (**R**). Lower recognition of anti-Tn antibodies to human embryonic kidney HEK-293 cells (**S**), and negative staining was observed without the primary anti-Tn antibodies (**T**).

**Figure 5:**
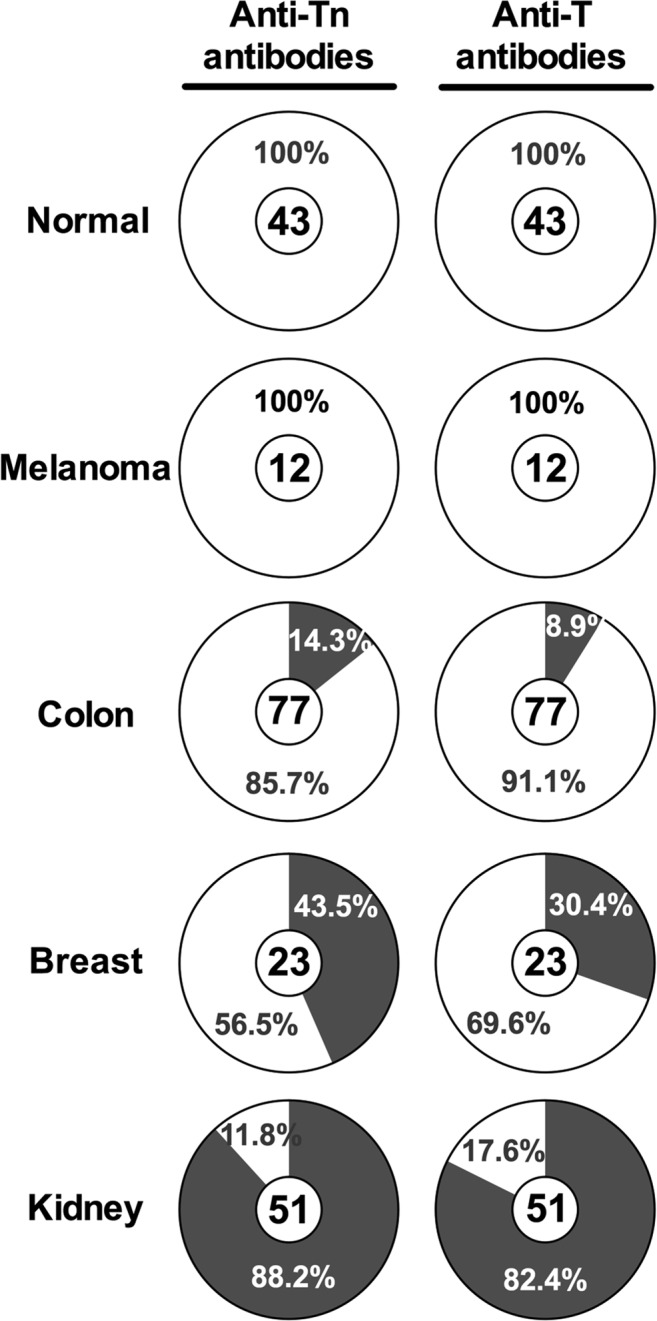
Recognition of purified antibodies to human tissues by TMAs. Summary of TMAs results for anti-Tn and anti-T antibodies. Percentage of non-recognized samples is shown as clear areas, whereas percentage of recognized samples is depicted as dark gray areas. Number of total samples for each tissue is noted inside each pie chart.

The capacity of human purified anti-Tn and anti-T antibodies to recognize human carcinoma tissues was analyzed by tissue micro-arrays (TMAs). Human TMAs from normal breast, kidney, colon, skin, placenta, cervix, fallopian tube, pancreas, amygdale, endometrium, small intestine, ovary, spleen, and brain tissues, as well as carcinomas from skin, colon, breast, and kidney tissues were assayed to study carcinoma recognition of purified antibodies. Representative images of human normal and malignant tissues, and human cell lines with negative and positive staining are shown in Fig. 4. Purified anti-Tn and anti-T antibodies did not recognize (0/42) normal human tissues (Fig. 5). Human melanoma tissues were not recognized (0/12) by purified anti-Tn or anti-T antibodies. Human carcinoma colon tissues were positively stained in 14% (11/77) by anti-Tn antibodies and in 8.9% (7/79) by anti-T antibodies. In addition, breast carcinoma tissue showed positive immunoreactivity in 43% (10/23) and 30% (7/23) by anti-Tn and anti-T antibodies, respectively. Table 5 shows features of human breast carcinomas. Also, human breast tumor T47D cell line showed strong positive recognition by purified antibodies (Fig. 4). Human embryonic kidney (HEK-293) cells were less immunoreactive that tumor T47D cells with purified anti-Tn and anti-T antibodies (Fig. 4). Human kidney carcinoma was the most recognized tissue by these antibodies, showing positive staining in 88% (45/51) by anti-Tn antibodies and in 82% (42/51) of carcinoma tissues by anti-T antibodies (Fig. 5). Table 6 shows characteristic of human kidney tumor tissues and the recognition of purified antibodies.

**Table 5:** Characteristics of analyzed human breast tissues and recognition of purified antibodies.

Histology	Surgery	Tumor size (cm)	Metastasis/analyzed node	Fuhrman grade	Anti-T recognition (score, assessment)	Anti-Tn recognition (score, assessment)
Invasive ductal carcinoma	Quadrantectomy	2.0	1/10	2	0, negative	0, negative
Lobular carcinoma	Mastectomy	4.0	8/12	4	1, negative	1, negative
Invasive ductal carcinoma	Quadrantectomy	2.5	0/10	3	3, positive	3, positive
Invasive ductal carcinoma	Quadrantectomy	2.5	0/1	3	0, negative	0, negative
Invasive ductal carcinoma	Mastectomy	3.8	0/14	2	2, positive	3, positive
Invasive ductal carcinoma	nd	5.0	1/1	2	3, positive	2, positive
Invasive ductal carcinoma	Mastectomy	9.0	nd	3	1, negative	0, negative
Invasive ductal carcinoma	Mastectomy	1.8	nd	3	1, negative	3, positive
Invasive ductal carcinoma	Mastectomy	3.6	1/11	2	2, positive	3, positive
Lobular carcinoma	Mastectomy	4.0	0/8	2	1,negative	2, positive
Invasive ductal carcinoma	Mastectomy	nd	6/6	2	0, negative	2, positive
Invasive ductal carcinoma	Mastectomy	4.0	nd	3	0, negative	0, negative
Invasive ductal carcinoma	Quadrantectomy	2.0	nd	2	0, negative	0, negative
Invasive ductal carcinoma	Mastectomy	6.0	6/6	3	0, negative	1, negative
Invasive ductal carcinoma	Quadrantectomy	4.0	nd	3	3, positive	3, positive
Invasive ductal carcinoma	nd	4.0	nd	3	1, negative	2, positive
Lobular carcinoma	Quadrantectomy	2.5	0/6	2	0, negative	0, negative
Invasive ductal carcinoma	Mastectomy	2.6	0/12	2	0, negative	0, negative
Invasive ductal carcinoma	Mastectomy	2.0	0/14	2	0, negative	1, negative
Invasive ductal carcinoma	Quadrantectomy	2.0	nd	3	0, negative	0, negative
Invasive ductal carcinoma	Quadrantectomy	1.5	0/14	3	2, positive	1, negative
Invasive ductal carcinoma	Quadrantectomy	nd	nd	3	0, negative	0, negative
Invasive ductal carcinoma	Mastectomy	2.5	0/3	1	2, positive	2, positive

nd: not determined.

**Table 6:** Characteristics of analyzed human kidney tissues and recognition of purified antibodies.

Histology	Fuhrman grade	Anti-T recognition (score, assessment)	Anti-Tn recognition (score, assessment)
Clear cell renal carcinoma	3	0, negative	0, negative
Clear cell renal carcinoma	2	0, negative	0, negative
Clear cell renal carcinoma	2	1, negative	2, positive
Clear cell renal carcinoma	3	2, positive	3, positive
Clear cell renal carcinoma	2	3, positive	3, positive
Clear cell renal carcinoma	2	2, positive	3, positive
Clear cell renal carcinoma	2	0, negative	3, positive
Clear cell renal carcinoma	2	2, positive	3, positive
Clear cell renal carcinoma	4	3, positive	3, positive
Clear cell renal carcinoma	2	2, positive	2, positive
Clear cell renal carcinoma	1	3, positive	3, positive
Clear cell renal carcinoma	3	2, positive	3, positive
Clear cell renal carcinoma	2	0, negative	0, negative
Type 1 papillary renal cell carcinoma	2	3, positive	3, positive
Clear cell renal carcinoma	3	1, negative	1, negative
Clear cell renal carcinoma	3	3, positive	3, positive
Clear cell renal carcinoma	2	2, positive	3, positive
Type 2 papillary renal cell carcinoma	2	2, positive	3, positive
Oncocytic renal tumor cells	2	2, positive	2, positive
Clear cell renal carcinoma	2	3, positive	3, positive
Clear cell renal carcinoma	2	3, positive	3, positive
Clear cell renal carcinoma	2	3, positive	2, positive
Clear cell renal carcinoma	2	3, positive	3, positive
Clear cell renal carcinoma	2	0, negative	2, positive
Clear cell renal carcinoma	2	3, positive	3, positive
Clear cell renal carcinoma	2	3, positive	3, positive
Clear cell renal carcinoma	3	3, positive	3, positive
Clear cell renal carcinoma	2	0, negative	0, negative
Clear cell renal carcinoma	2	3, positive	3, positive
Type 1 papillary renal cell carcinoma	1	3, positive	3, positive
Clear cell renal carcinoma	3	3, positive	3, positive
Clear cell renal carcinoma	2	3, positive	3, positive
Clear cell renal carcinoma	2	3, positive	3, positive
Clear cell renal carcinoma	2	3, positive	3, positive
Clear cell renal carcinoma	3	3, positive	3, positive
Clear cell renal carcinoma	2	3, positive	3, positive
Clear cell renal carcinoma	2	3, positive	3, positive
Clear cell renal carcinoma	2	2, positive	2, positive
Clear cell renal carcinoma	2	3, positive	3, positive
Clear cell renal carcinoma	3	3, positive	3, positive
Clear cell renal carcinoma	2	2, positive	3, positive
Clear cell renal carcinoma	2	3, positive	3, positive
Clear cell renal carcinoma	2	0, negative	0, negative
Clear cell renal carcinoma	2	3, positive	3, positive
Clear cell renal carcinoma	2	3, positive	3, positive
Clear cell renal carcinoma	2	3, positive	2, positive
Clear cell renal carcinoma	3	3, positive	3, positive
Clear cell renal carcinoma	3	3, positive	3, positive
Type 2 papillary renal cell carcinoma	2	2, positive	2, positive
Clear cell renal carcinoma	3	3, positive	2, positive
Clear cell renal carcinoma	2	3, positive	3, positive

**Figure 6:**
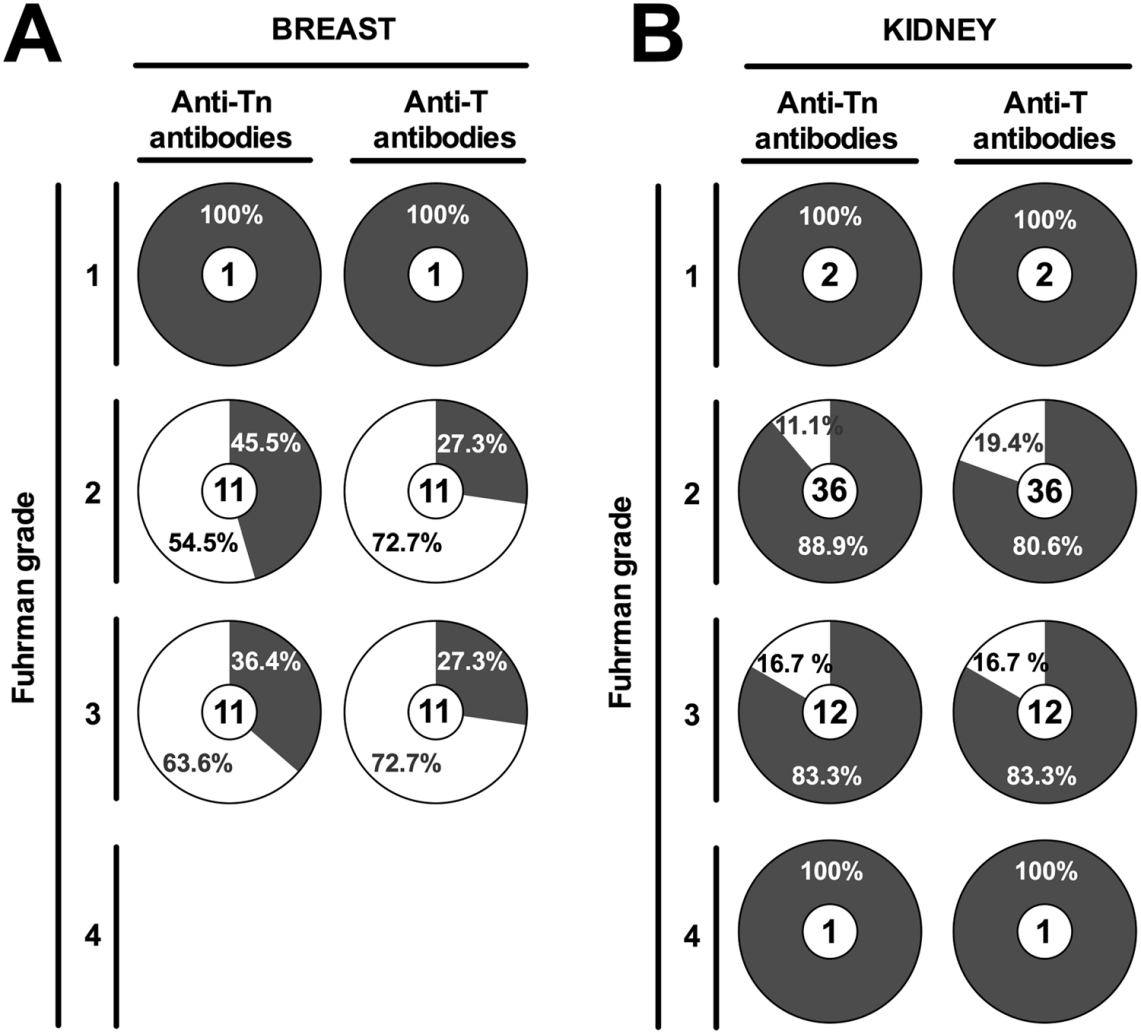
Positive recognition of purified anti-Tn and anti-T antibodies in relation to carcinoma malignancy state (Fuhrman grade). Summary of TMAs results for anti-Tn and anti-T antibodies for breast (**A**) and kidney (**B**) human carcinoma tissues. Percentage of non-recognized samples is shown as clear areas, whereas percentage of recognized samples is depicted as dark gray areas. Number of total samples for each tissue is noted inside each pie chart. Fuhrman grade (1–4) is indicated to the left.

Positive recognition of purified anti-Tn and anti-T antibodies was analyzed in relation to carcinoma malignancy state (Fuhrman degree classification). Anti-Tn antibodies identified 45% (5/11) degree 2 human breast carcinomas, and 36% (4/11) degree 3 (Fig. 6A). Degree 2 human breast carcinoma tissues showed 27% (3/11) positive recognition by anti-T antibodies, and identical result was the recognition (3/11) of degree 3 breast carcinoma tissues. Kidney carcinoma TMAs revealed that anti-Tn antibodies identified 89% (32/36) degree 2 and 83% (10/12) degree 3 carcinoma tissues, whereas anti-T antibodies recognized 80% (29/36) degree 2 and 83% (10/12) degree 3 carcinoma tissues (Fig. 6B).

## Discussion

Passive immunotherapy of patients with cancer pathologies implicate incorporation of molecules addressed against the tumor. Thus, specific molecules are used to decrease the transformation, proliferation and survival of cancer cells. Mouse monoclonal humanized antibodies (mAbs) such as obinutuzumab, rituximab and trastuzumab are examples of successful passive immunotherapies in the treatment of cancer patients^20^. However, these mAb treatments showed side effects such as ocular and neuro-ophthalmic toxicity, skin toxicities with different severities (from mild lichenoid reaction to severe toxic epidermal necrolysis), increased risk factors in development of hypogammaglobulinemia and infections (post-rituximab), and increased cumulative incidence of breast cancer brain metastases after HER2-directed therapy (trastuzumab)^21–24^. In the present work, we obtained human anti-Tn and anti-T antibodies that specifically recognize human carcinoma tissues, with potential use for restoration of anti-glycan antibody concentrations of patients presenting epithelial tumor pathologies.

Immobilized ASF and OSM allowed the purification of natural antibodies present in normal human plasma. Both purified antibodies enhanced more than 120-times the purification degree compared to plasma presence. Related to immunoglobulin isotype of purified antibodies, anti-ASF antibodies were mainly IgG whereas anti-OSM antibodies contained IgM in similar proportion to IgG. In agreement with this purification, the presence of IgM and IgG2 subclass as constitutive of natural anti-glycan antibodies has been frequently described^25,26^.

The classical way to study glycan recognition of proteins is through competitive assays with soluble carbohydrates. This useful tool allowed us to observe that, upon purification, human natural anti-ASF antibodies recognize related Thomsen-Friedenreich structure (T antigen), and that human natural anti-OSM antibodies recognize Tn antigen. Thus, both purified antibodies showed the expected glycan recognition specificity.

The ability of these purified anti-glycan antibodies to recognize human carcinomas was studied using TMAs. Purified anti-T and anti-Tn antibodies did not interact with normal human tissues, a consistent observation since these antibodies were purified from normal individuals. This represents an advantage compared to observations from previous antibody approaches: therapeutic use for several mouse humanized monoclonal antibodies reported side effects as skin and neuro-ophthalmic toxicities, thus suggesting recognition of normal tissues^23,24^.

Purified anti-Tn and anti-T antibodies did not show capacity to recognize human melanomas, and colon carcinomas were poorly recognized. The binding ability of these antibodies improved for breast human carcinomas tissues (43% for anti-Tn antibodies and 30% for anti-T antibodies), and they were even more efficient in recognizing human kidney carcinoma tissues. Trastuzumab (anti-Her2 antibody) is present in the pharmaceutical market having only 3% Her2-positive immunostaining across human carcinomas, with frequencies ranging from 0.4% in hepatocellular carcinoma to 12.4% in bladder cancer^27^. The antibodies obtained herein have better recognition ability to human carcinomas than trastuzumab, thus deserving further attention.

The analysis of carcinoma Fuhrman grades related to positive recognition for the obtained antibodies showed no differences. Anti-Tn and anti-T antibodies recognized equally well carcinomas with Fuhrman degree 2 and 3 from human breast and kidney tissues. This fact shows that these antibodies could be useful for patients’ treatment with early Fuhrman grades of breast and kidney carcinomas, such as degree 2.

In summary, we have outlined the production of two natural antibodies from normal human plasma. Their relevant capacity to efficiently and specifically recognize human carcinoma tissues brings hope about their potential use as antitumoral antibodies to restitute the anti-Tn and anti-T antibody concentrations in patients with carcinoma pathologies.

## Methods

### Human plasma

Normal human plasma samples from healthy donors were collected by Laboratorio de Hemoderivados, Universidad Nacional de Córdoba (UNC), Argentina. Written informed consent was obtained from every donor. All plasma demonstrated negative serology tests for common infectious diseases. A plasma pool from 100 healthy donors was used as starting material for antibody purification. All procedures were approved by the Ethics Committee of CIQUIBIC-CONICET (UNC). All experiments were performed in accordance with Ethical Guidelines on Research Involving Human Subjects^28^.

### Antibody purification

Human antibodies were purified by affinity chromatography using asialofetuin (ASF; Sigma-Aldrich, St. Louis, MO, USA) or ovine submaxyllary mucin (OSM; Accurate Chemical & Scientific Corporation, Westbury, NY, USA) immobilized in cyanogen-bromide-activated Sepharose 4B column (Sigma-Aldrich, St. Louis, MO, USA). Gammaglobulin fraction from pooled human plasma were loaded into ASF- or OSM-Sepharose column, washed with 0.1 M Tris-HCl (pH 8.0) and 0.01 M Tris-HCl (pH 8.0), and eluted with 0.2 M glycine-HCl (pH 2.5). Specific antibodies were recovered in 1 M Tris (pH 8.0). Affinity-purified antibodies were dialyzed against PBS and stored at −18 °C until use.

### SDS-PAGE and western blotting

Homogeneity of purified antibodies was confirmed by SDS-PAGE and Coomassie Brilliant Blue staining^29^. To evaluate their isotypes, purified antibodies were subjected to SDS-PAGE, and electrotransferred membranes were blocked with 1% skim milk in PBS for 60 min at room temperature (RT). HRP-labeled IgG anti-human IgG, anti-human IgM, or anti-total immunoglobulin antibodies (Sigma-Aldrich, St. Louis, USA; 1/1000 dilution in 0.05% Tween 20 in PBS) were incubated for 2 h at RT and washed with PBS. Color reaction was developed using 0.1 mg/ml 4-chloro-1-naphthol and 0.02% H_2_O_2_ in methanol-TBS for 20 min, and stopped by washing with distilled water.

### Measurement of total proteins and immunoglobulins

A bicinchoninic acid assay was used to measure total proteins with BSA as the standard (Pierce, Thermo Scientific). To measure total immunoglobulin concentration, wells of microtiter plates were coated with several concentrations of purified total human immunoglobulins (Gammaglobulina-T, Laboratorio de Hemoderivados, UNC, Argentina) in 0.1 M carbonate buffer (pH 9.5) overnight at 4 °C. After blocking with PBS containing 1% BSA and 0.05% Tween 20, plates were incubated with 1:1000 HRP-conjugated IgG anti-human total immunoglobulin antibodies (Sigma-Aldrich, St. Louis, USA) in PBS-T, for 2 h at RT. Plates were washed 4x with PBS-T, and color reaction was developed using 2 mg/ml o-phenylenediamine and 0.02% H_2_O_2_ in 0.1 M sodium citrate (pH 5.0), at RT for 10 min. Reaction was stopped by addition of 0.5 M sulfuric acid. Absorbance values were read at 490 nm with a microplate reader. Similarly, human IgM and IgG was measured using HRP-labeled IgG anti-human IgM and anti-human IgG antibodies, and concentrations determined from standard curves of human IgG and IgM, respectively. Radial immunodiffusion for measuring human IgG1, IgG2, IgG3 and IgG4 was performed as suggested by manufacturer (The Binding Site Inc., San Diego, USA) and the concentrations were determined from standard curves.

### Antigen recognition of antibodies

Antigen recognition of antibodies was measured by ELISA. Wells of microtiter plates were coated with antigen (ASF or OSM) in 0.1 M carbonate buffer (pH 9.5) overnight at 4 °C. After blocking with PBS containing 1% BSA and 0.05% Tween 20, we incubated plates with various dilutions of purified antibodies in PBS-T, for 2 h at RT. Plates were washed 4x with PBS-T, then incubated with 1:1000 diluted HRP-conjugated IgG anti-human total immunoglobulin antibodies for 1 h at RT. Plates were washed 4x with PBS-T, and color reaction was developed as previously described.

### Antibody specificity by competitive ELISA

Steps of competitive ELISA were developed similarly to previous item, except that the optimal antibody dilution was preincubated with each carbohydrate for 1 h before addition to wells. The optimal antibody dilution showing ∼1.0 optical density against the target antigens was assayed. Results were expressed as the percent inhibition caused by the competition of soluble sugar presence.

### Cell lines

Human breast adenocarcinoma (T47D), and human embryonic kidney (HEK-293) cells were from CIQUIBIC-CONICET/Dept. of Biological Chemistry, Universidad Nacional de Córdoba, Argentina. Human cell lines were grown at 37 °C in an incubator (10% CO_2_ atmosphere) in Dulbecco’s modified Eagle’s medium (DMEM) (Sigma-Aldrich, St. Louis, MO) supplemented with 10% fetal calf serum (FCS), 1 mM sodium pyruvate, and nonessential amino acids.

### Tissue microarrays (TMAs)

All procedures, performed in accordance with Ethical Guidelines on Research Involving Human Subjects^28^ and with ethical standards as laid down in the 1964 Declaration of Helsinki and its later amendments, were approved by the Ethics Committee of CIQUIBIC-CONICET; informed consent was obtained from the patients. Paraffin-embedded tissues were selected from histological file and used in a blind-retrospective manner. Representative areas from normal tissues (n = 42), skin melanoma (n = 12) and carcinomas (colon n = 77, breast n = 23, kidney n = 51) were carefully marked on H&E-stained sections. Tissue cores of 2 mm diameter were obtained from each specimen and were precisely arrayed into a new paraffin block. 4 μm thickness sections of this new block were cut and mounted on polarized slides to future staining.

### Immunohistochemistry

Paraffin sections were dewaxed in a xylene-ethanol series. Endogenous peroxides were removed by methanol 3.5% hydrogen peroxide incubation at room temperature for 30 minutes. Heat-induced epitope retrieval was performed with a 0.1 M sodium citrate buffer (pH 6.0). A 1-hour blocking step with 10% goat serum was done prior to adding primary antibody followed by HRP-conjugated anti-human total immunoglobulin antibodies (Sigma-Aldrich). Detection was performed using 3,3′-diaminobenzidine and counterstained with Biopur hematoxylin. Sections were then dehydrated through a series of ethanol to xylene washes and cover slipped with Canada Balsam. Images of representative tissue spots were taken at 40× magnification with a Leica microscope.

### Criteria for classification of staining patterns

Scores were defined in terms of staining pattern. **Score 0**: No observable staining, or membrane staining that is incomplete and is faint/barely perceptible in ≤10% of invasive tumor cells. **Score 1**: Incomplete membrane staining that is faint/barely perceptible in ≤10% of invasive tumor cells. **Score 2**: Circumferential membrane staining that is incomplete and/or weak/moderated in >10% of invasive tumor cells, or complete and circumferential intense membrane staining in ≤10% of invasive tumor cells. **Score 3**: Homogeneous, dark, circumferential (chicken wire) pattern in >10% of invasive tumor cells.

Scores 0 and 1 were assessed as negative, whereas scores 2 and 3 were assessed as positive.
